# Influential children in middle childhood peer culture: Effects of temperament and community culture

**DOI:** 10.3389/fpsyg.2022.923469

**Published:** 2022-10-21

**Authors:** Roy P. Martin, Audra Michele Lease

**Affiliations:** Department of Educational Psychology, University of Georgia, Athens, GA, United States

**Keywords:** temperament, childhood, influence, culture, status

## Abstract

For children in middle childhood, the social world, particularly the behavior and attitudes of their school peers, has been shown to be an important factor in their educational and mental health outcomes. In the school environment, some children seem to influence the attitudes and behavior of their peers more than others. The behavior patterns of children, as reflected in temperamental traits, have been shown to drive peer perception in important ways and might play a role in identifying the individuals and social processes that operate in peer influence. It seems likely that temperamental traits will have different effects on school peers, dependent on characteristics of the school attended. Fourth and fifth grade children from four rural counties in the southeastern portion of the United States were studied. Temperamental characteristics were assessed based on teacher perception of six characteristics. Peer perceptions of the extent to which each child was perceived to influence others in five areas of school culture (e.g., academics, sports) was measured through a peer nomination procedure. Additional status-related perceptions and behaviors of participating children were also assessed by peer nominations. Teacher ratings of temperamental behaviors were submitted to latent profile analyses resulting in a seven-cluster model. Results indicated temperamental profiles were significantly and meaningfully associated with peer perceptions of influence as well as social status. Further, demographic differences between two groups of schools were found to moderate the effects that temperament profile had on peer influence.

## Introduction

Children in middle childhood are acutely attentive to their social world. They have emerged from living in a world of adult caretakers (e.g., parents and teachers) into the complex social world of peers. A good proportion of the social interaction with peers takes place in schools where children must learn to adapt to a staggering array of individual differences in behaviors, attitudes, and expectations. How the child copes with this social environment has important consequences; it can determine acceptance into subgroups (e.g., cliques; Gazelle and Ladd, 2003; Rubin et al., 2006), as well as general social status, mental health, and academic achievement (Asendorpf, 1990; Rubin et al., 2010; Rubin and Coplan, 2010; Masland and Lease, 2016). One important aspect of the social life of children in middle childhood is that peers, through a variety of means, influence the behavior and attitudes of one another. Peer influence during the late elementary school years has been shown to effect aggressive-disruptive behavior (Powers and Bierman, 2013) as well as academic engagement (Gremmen et al., 2018). Peer influence can stem from close friends but also from the broader peer group (Gottman and Mettetal, 1986). Identifying which children are most likely to be influential, and the social circumstances in which children are influential, is an important question and the focus of the current study. The Dominance-Prestige model of social influence has guided our thinking about how temperament might affect the influence one child has on another. This model posits that there are two pathways that can be used to climb the social hierarchy (Strayer and Trudel, 1984; Cheng et al., 2013; Maner, 2017). The Dominance pathway is established in the context of agonistic exchanges using manipulation and aggressive strategies. It seems likely that children who exhibit higher levels of the temperamental trait labeled irritability or negative emotionality are likely to rely on dominance and antagonistic behaviors to establish influence. The Prestige path, in contrast, is accomplished based on skills, knowledge, and abilities. Those who have higher status based on prestige are perceived as having higher competence and altruistic tendencies. The temperamental characteristics most associated with altruistic behaviors are positive emotionality. Skills and abilities most pertinent in elementary school are academic and athletic abilities. Academic ability is associated with temperamental traits related to self-regulation of attention, which, in turn (Martin, 1989), is strongly linked to school performance (Martin, 1989; Martin et al., 2020). Social skills are related to the temperamental traits of sociability and inhibition (Rubin et al., 2010), and having a high level of gross motor vigor (activity level) is logically related to athletic ability. Temperament research has traditionally focused on the measurement (Rothbart et al., 2001; Halverson et al., 2003; Putnam and Rothbart, 2006) and structure of early appearing individual differences of children (Beekman et al., 2015; Martin et al., 2020), the extent to which they are genetically linked (Saudino and Wang, 2012; Tackett et al., 2013; Scott et al., 2016) as well as the extent to which they are associated with a variety of physiological functions (Kagan et al., 1988; Van Ijzendoorn et al., 2012; White et al., 2012; Marsman et al., 2013). In addition, there has been considerable effort to demonstrate that temperament traits are related to a wide range of behaviors in childhood, including diagnosed mental health problems (Thomas and Chess, 1977; Tackett et al., 2013). Among the most provocative research efforts are those that demonstrate the long-range effects of temperament in early childhood on adult attitudes and behaviors including political orientation (Block and Block, 2006), adult personality (Caspi and Silva, 1995), adult psychiatric disorders (Caspi et al., 1996), antisocial behavior in adulthood (Henry et al., 1996; Moffitt et al., 2002), and gambling (Slutske et al., 2012). There has been much less attention on temperament effects on schooling. The research that has been published has primarily related temperament to achievement and behavior problems (Martin and Holbrook, 1985; Martin, 1989; Nelson et al., 1999; Guerin et al., 2003) as well as the management of individual differences in the classroom (McClowry, 2014). There has been a notable lack of research on temperament as it affects social relationships in general and peer influence, in particular. Given the importance to life-span development of early educational experiences, this is an unfortunate oversight. One recent study by Martin et al. (2020) has addressed the issue of the relationship between peer influence and temperament. This research has shown that the temperamental profiles of children as assessed by parents and teachers are meaningfully related to the influence children have on one another in elementary school. However, this research did not address the issue of the effects of different macro-social environments on this relationship. The purpose of the current study was to refine aspects of the prior research and to directly address the effect of the broad social environment in which children live on temperament-influence relations. Three questions will be addressed in this paper: First, how do temperament-based profiles based on teacher perception relate to the influence peers have on one another as reported by the peers themselves? While this question was addressed in the prior research, the sample analyzed has been changed. The current sample is composed exclusively of 4^th^ and 5^th^ grade students, while the previous sample included 3^rd^ graders. This sharpens the focus of the research on late elementary school. Second, the profile model in the current analysis focuses exclusively on teacher perceptions of temperament and does not include data from parents as was the case in prior analyses. Third, profile models used in the prior research included parental and teacher perception of academic ability (intelligence). The current research focuses exclusively on tradition temperament constructs in the development of profile models. In addition, several refinements are made in the current analysis to help control for gender factors in the peer nomination procedures as well as to control differences among schools in the way that peer nominations were done. When the best fitting temperament profile model has been developed and associations to peer influence determined, the second question to be addressed becomes, what social status and status-related behavioral characteristics are most strongly associated with the temperament profiles of influential children. This analysis is designed to set the stage for future researchers to determine the longitudinal pathways operating from temperamental characteristics and social status characteristics to influence. The characteristics investigated include peer perception of popularity, likability, aggression, a tendency to be sympathetic, to work hard in school, to be perceived as cool, and to be good at sports. The characteristics were selected to present aspects of the dominance versus prestige approach to status attainment. The third question to be addressed relates to the effect of the broader social-cultural environment of the schools studied in modifying the association between temperament profiles and influence. The specific question that is addressed is: Are the temperamental characteristics of influential children in schools located in counties with higher high school graduation rates in the adult population different from the temperamental characteristics of influential children in schools located in counties with lower high school graduation rates?

## Materials and methods

### Participants

Lease and colleagues (Kwon and Lease, 2014; Lease et al., 2020) initiated two different data collections designed to compare a variety of social and education outcomes from schools in the southeastern portion of the United States. The children studied included those attending schools in rural and semi-rural counties. The data were collected from rural areas to truncate socio-economic differences within schools which have been shown to relate to a range of schooling outcomes. From this larger project, the data analyzed for the current study were selected to maximize the similarity in age and gender distribution of the children in two groups of schools. The groups of schools were differentiated by demographic characteristics, particularly the education level of the population from which the students were drawn. Data in the current analysis were obtained from teachers and students in six schools in three counties (School Group A: 22 teachers, and 448 students) and four schools in one county (School group B; 24 teachers, 349 students). All children were enrolled in the 4th or 5th grades, and all were between 9 and 12 years-of-age. Table 1 presents the demographic characteristics of the participants in Group A and in Group B schools as well as the total sample. The data in Table 1 indicate that the samples were similar except of the racial/ethnic composition; Group A school served a more diverse group of students.

**Table 1:** Demographic characteristics of children in two school groups.

Cohort	School group A		School group B		Total sample	
	*N*	%	*N*	%	*N*	%
Sex						
Female	237	52.9	185	53.0	422	52.9
Male	211	47.1	164	47.0	375	47.1
Grade						
4	162	36.2	153	43.8	315	39.5
5	286	63.8	196	56.2	482	60.5
Age						
9	71	15.8	45	12.9	116	14.6
10	162	36.2	144	41.3	306	38.4
11	195	43.5	145	41.5	340	42.7
12	20	4.5	15	4.3	35	4.4
Race/ethnicity						
Black	185	41.0	49	14.1	234	29.4
White	249	55.6	267	76.9	516	64.7
Other	14	3.1	33	9.0	47	5.6
Total	448		349		797	

### Demographic characteristics of counties in which schools were located

To help understand the cultural context in which the students lived, we obtained data at the county level in which each school was situated. Data were obtained from the US census for 2010. All four counties were rural with no cities of population greater than 4,000. Between 2000 and 2010, School Group B was in a county that had significantly gained population, while the three counties in which Group A schools were located had lost population. The racial/ethnic composition of the public schools in Group A were more racially/ethnically diverse (44.4% minority children) than the county containing Group B schools (23.0% minority children). The populations in the county served by Group B schools had a higher median family income ($49,700) than the counties served by Group A schools ($38,033), but both were significantly below the United States median family income level in 2010 ($62,664). Both sets of schools served populations with very similar levels of educational attainment. For example, the percentage of the population 25 years or older who did not graduate from high school or obtain a GED was 16.9% (Group A) and 16.8% (Group B), but both were above the national average of 11.6%. However, the minority population was significantly less affluent and had lower mean educational attainment than the White population in all counties. Educational attainment differences were particularly lower for minority males. In the counties containing the Group A schools, the mean percentage of minority males (25 years and older) who did not graduate from high school or have a GED was 40.9%, while in the county containing the Group B schools, this percentage was 19.8%. In summary, both groups of schools were in rural areas in which the median family income was lower than the national average as was the educational attainment for the adult population. However, the minority population was far less affluent and less formally educated than the White population of these counties. Group A schools were in a county with a much higher proportion of minority residents than was the case for Group B schools. Thus, children in Group A schools grew up in an environment in which the adults, particularly males, were less educated and had fewer material resources. This was particularly true of the minority children in Group A.

### Study procedure

For the original data collection from which the current participants were selected, approval was obtained from the superintendent of the school district. Then, individual school principals and staff were contacted; only one school declined participation. Active parental consent for student participation was required, and child assent was obtained prior to the administration of questionnaires. The roster of students used for peer nominations included only the names of students who had obtained parental consent to participate. Nonparticipating students were given the option of working quietly at their desks. All procedures were approved by a university Institutional Review Board.

### Measurement

#### Teacher perception of temperament

Teacher perceptions of their students’ temperament characteristics were assessed based on a modified version of the Individual Differences of Children and Adolescents questionnaire (ICID; Halverson et al., 2003). The ICID was designed for parents; the revised form for teachers was modified to make it appropriate for classroom teachers. The measure was an abbreviation of the ICID and was very similar in length and item content to a published abbreviated version of the ICID (Deal et al., 2007). Seven scales from the Teacher ICID measure were used in the current study to develop temperament profiles. These scales were designed to measure classic temperamental traits. Inhibition and fearfulness were combined because they were highly correlated (0.80). This resulted in six temperament scales. The internal consistency reliability as indexed by the alpha coefficient for the 4^th^ and 5^th^ grade children studied in this analysis were as follows: activity level (alpha = 0.80), sociability (alpha = 0.90), positive emotionality (alpha = 0.88), negative emotionality (alpha = 0.93), distractibility (distractibility alpha = 0.81), and inhibition (inhibition and fearfulness, alpha = 0.80). The concurrent validity of the teacher form of the ICID has been documented through scale and profile similarities to parental ratings on the same instrument, as well as to important outcomes for children in elementary school such as behavior problem ratings and academic achievement (Martin et al., 2020).

#### Peer perceptions

Peer perception of influence was measured by self-report measure based on existing scales and/or theoretical formulation by Hawley et al. (2002), Keltner et al. (2001), and Janes and Olson (2000); see (Lease et al., 2020), for a complete description of these procedures. Influence was assessed in five areas: academics, sports, peer cultural trends (e.g., clothing, music), make-believe games, and inappropriate behavior (e.g., talking back to the teacher; fooling around when the teacher leaves the room). These measures resulted in six indicators of influence for each child, one for each of the five areas of influence and a total influence score created by summing scores across all five areas. An example of the questions used to elicit peer nominations of influence is: “Think of a time when you decided to work really hard on a class project or study hard for a test because other kids were. What kids made you want to study hard, too”? From a listing of consented children provided to each student, they recorded the number of the children who fit this description. In some schools, children were asked to nominate peers from their class (homeroom), whereas in other schools, children were nominated from the grade level. The numbers of nominations children received were standardized (*M* = 0, SD = 1) at the classroom level or at the school level, depending on which procedure was used. Standardization was used to control for the differing number of nominations possible based on the number of participating peers in the classroom or grade level. In addition, standardization was carried out separately for girls and boys. Gender plays a role in many aspects of peer relationship, particularly in middle childhood. Children interact with same-gender peers more often than opposite-gender peers (Martin et al., 2013). To better understand the characteristics of children who were considered most influential, children were asked to nominate children who fit several behavioral or status characteristics. These nomination procedures were based on similar measurement procedures by Parkhurst and Hopmeyer (1998) and Coie et al. (1982). From these descriptions, seven scores were created, following the same standardization procedures described for influence nominations (above). Children were asked to nominate the peers they would most like to play with and those they would least like to play with. A social preference score was derived by subtracting the standardized least liked score from the standardized most liked score (Coie et al., 1982). A similar process was used to obtain a measure of popularity; that is, least popular scores were subtracted from the most popular scores. Further, nominations were obtained for the children who were perceived as ‘cool’ and well known in the school. Finally, nominations were obtained in response to the following: This person tries hard to do good schoolwork (tries hard); this person shows sympathy to a peer who is sad, hurt, or upset (shows sympathy); and this person is good at sports (good athlete). A final set of five descriptors indicating the tendency to be aggressive were obtained from peers and were aggregated into one score. Examples of these items are: “This person makes mean faces at someone when they are upset with them” and “This person overreacts and is easily pushed to anger.” The five-item aggression scale had a coefficient alpha of 0.92.

### Statistical procedures

Children were given a score on each of the six temperamental characteristics rated by teachers. These scores were standardized for each teacher/classroom. This procedure helped to control for teacher biases in rating student behavior. These scores were submitted to a latent profile analysis using Mplus (Muthen and Muthen, 1998–2012). This type of analysis assumes that within a large group of children, there are subgroups (clusters) who share common patterns or profiles of characteristics, and that these profiles describe the children more accurately than any of the individual characteristics. These subgroups occur because there are correlations among the behavioral traits used as indicators of the subgroups. A latent profile is a description of a group (cluster) of individuals that share a pattern of behavior. It is latent in the sense that it is not known by the researchers at the time of data collection or analysis. The goal of the analysis is to statistically determine the smallest number of latent clusters that is sufficient to account for the associations observed among the measured variables. The cluster of individuals within a profile is typically identified by their average score on each indicator variable. All the individuals within the group do not have the same score, but the scores of children in the group are more similar than to children in any other group. A central question in latent profile research is how many clusters best fit the data. It is customary to test a wide range of models to find the one that best fits the statistical criterion. Previous research indicates that from 3 to 9 clusters meet these criteria for temperament and related child behavioral measures (Asendorpf and van Aken, 1999; Martin et al., 2020). The criteria that are most often used include a decline in the three information criteria (Akaike, Bayesian, and Bayesian adjusted for sample size) as more clusters are added to the model. Some researchers (Morin and Marsh, 2015) plot these criteria across models and look for an elbow in the declining plot line. One other criterion that was used in the current analysis is the size of the smallest cluster. Since differences in two groups of school were to be investigated, a minimal cluster size of 30 children was established before the analysis was done (4.0% of the sample). Two simplifying assumptions are made to reduce the number of parameters that are estimated in the model. The first assumption is that the correlation among indicator variables in each profile is zero. This assumption is never exactly met, but in the current analysis, all variables were correlated < 0.30 in each profile. The second assumption is that the standard deviation of each variable is the same for all profiles. Modeling the effects of indicator correlations within profiles and standard deviation differences across profiles would require much large samples than were available in the current analysis. These assumptions are common practice (Muthen and Muthen, 1998–2012).

## Results

### Temperament profiles

Table 2 presents the outcomes of the latent class analyses for models containing 3 to 9 clusters. All three information criteria declined as the number of clusters in the model increased. The entropy index in all models was excellent. All the lowest mean classification probabilities were also excellent. Thus, these statistical indices were not particularly helpful in determining the best model fit. Consistent with suggestions by Maiano, et al. (2011) and Morin and Marsh (2015), when other indices do not point to a best fitting model, the rate of decline in the information criteria should be examined. At some point, as the number of clusters in the model is increased, the rate of decline in information criteria flattens out. In the current analysis, the rate of decline slowed between the 7- and 8-cluster models indicating that both models should be examined to determine if they fit other criteria (e.g., some cluster is very small; one model fits better with temperament research outcomes in the literature than another). After consideration of all criteria, the 7-cluster model was selected. Table 3 presents the mean temperament score for each profile cluster, the standard deviation of each variable within clusters, and the number of children in each cluster. The clusters in this paper will be identified by a number (1–7) and a brief description. The numbering of the clusters is arbitrary. The clusters have been numbers based on the number of children presenting each temperament profile from larges to smallest. Cluster 1 children are labeled ‘average’ (41.2% of the sample). All their scores are between +0.70 and − 0.70 standard deviations (the middle 50% of each scale distribution). Cluster 2 was labeled ‘average with low levels of expression of negative emotion’ (18.4%). These children are hypothesized to have high levels of self-regulation of negative emotion. Cluster 3 children are labeled ‘happy, social, and active, with strong self-regulation of negative emotional expression and attention (12.4%). One aspect of their self-regulation of negative emotion is that they are perceived to be uninhibited in new situation and have fewer fears than their peers. Children in Cluster 4 exhibit a similar profile to those in Cluster 3, but their self-regulation of negative emotion and attention are in the average range (10.0%). Cluster 5 children are labeled ‘Active, distractible, negative’ and their self-regulation of negative emotion and attention is hypothesized to be below average (7.3%). They are marginally more social and uninhibited/fearless than their peers. Cluster 6 and 7 children are perceived by teachers as being far less sociable and more inhibited than their peers. In addition, Cluster 6 children (6.0%) are also far less vigorous and physically active than their peers. Cluster 7 children (4.6%) have similar levels of social withdrawal and fearfulness to children in Cluster 6 but express more negative emotion and are more distractible than their peers to determine if demographic characteristics were related to temperamental profiles, chi square test for cross-tabulation analyses were calculated for profile by child grade, by gender, and by minority/majority status. No significant effects were found. The standardization procedures used in this research (described above) resulted in means of each school group (A and B) being very near zero with standard deviation near 1 for all temperament characteristics in both groups. Thus, there was no difference in temperament ratings by teacher in the two school groups. To check to see if the percentage of children in each profile was similar across the two groups of schools, a 2 (school groups) by 7 (temperament profiles) cross-tabulation was done, and the analyses indicated no significant association of profile proportions for the two school groups.

**Table 2:** Results of latent profile analysis: six temperament indicators.

Clusters	LL^1^	Para^2^	Akaike^3^	BIC^4^	ABIC^5^	Entropy^6^	Small^7^	Prob^8^
3	−5932.7	26	11,917	12,039	11,957	0.88	18.80%	0.87
4	−5822.6	33	11,711	11,866	11,761	0.85	10.7	0.76
5	−5714.4	40	11,509	11,696	11,569	0.85	8.4	0.81
6	−5614.7	47	11,323	11,543	11,394	0.87	4	0.83
7	−5552.5	54	11,213	11,466	11,294	0.85	4.6	0.81
8	−5492.4	61	11,107	11,392	11,199	0.85	4.3	0.81
9	−5441.9	68	11,019	11,338	11,122	0.83	3.3	0.8

*n* = 797.

1 Log likelihood.

2 Number of parameters estimated by the model.

3 Akaike information criterion.

4 Bayesian information criterion.

5 Bayesian information criterion adjusted for sample size.

6 Entropy is an index of cluster separation; > 0.80 is good.

7 The size of the smallest cluster; we set a cut off at 4.0% of the sample.

8 Of all clusters in the model, the one with the lowest mean classification probability; > 70 is good.

**Table 3:** Mean temperament scale score (*z* score) for Each Cluster: 7 cluster model.

Cluster	act^1^	soc	pos	neg	dis	inhfer	Cluster size	
							*n*	%
1	−0.28	−0.35	−0.34	0.35	0.31	0.39	328	41.2
2	−0.39	−0.05	0.32	-0.95 ^2^	−0.59	−0.23	147	18.4
3	**1.15** ^2^	**1.57**	**1.70**	−1.19	−1.27	−1.32	99	12.4
4	**1.06**	**1.04**	**0.64**	0.07	0.33	−0.62	80	10.0
5	**0.81**	0.37	−0.76	**1.51**	**0.72**	−0.50	58	7.3
6	−1.59	−1.70	−0.73	−0.32	0.30	**1.61**	48	6.0
7	−0.59	−1.56	−2.09	**1.79**	**0.77**	**1.00**	37	4.6
Variances^3^	0.45	0.25	0.27	0.32	0.31	0.43		

1 act, activity level; soc, sociability; pos, positive emotionality; neg, negative emotionality; dis, distractibility; inh/fer, inhibition/fearfulness.

2 Means in bold are + 0.70 SD and means underlined are − 0.70 SD. These means are highlighted simply to aid the reader in seeing the primary characteristics that differentiate one cluster from another.

3 Variances around the mean for each temperament score is assumed to be the same for all profiles.

*N* = 797

### Temperament and peer influence

To determine the relationship between temperament profiles and influence, the total influence score was entered into a general linear model univariate analysis of variance as the dependent variable and temperament profiles were entered as the independent variables (using SPSS version 28). There was a significant effect for cluster (*F* = 17.07; *df = 6; p* < 0.001). The *R*^2^ of 0.109 indicated that about 11% of the variance in peer perceived total influence was associated with temperament profiles. Children in Cluster 6 (withdrawn, fearful, and low activity level) had the lowest average influence score, while children in clusters 1 and 2 (average, and average with low levels of negative emotionality) and 7 (withdrawn, with poor self-regulation of negative emotion and attention) had near average influence scores. The three most influential groups were children in Cluster 3 (happy, social, active, and with strong self-regulation), Cluster 4 (happy, social, active, and with average self-regulation), and Cluster 5 (active, distractible, and negative), and of these three clusters, children in Cluster 5 were perceived to have the most influence on their peers. A *post-hoc* analysis using the Gabriel method (see Table 4) indicated that there were three statistically different (alpha set at *p* < 0.05) homogeneous subgroups of clusters with Cluster 5 being most influential and Cluster 6 being least. All other clusters were not significantly different from one another. Because children with different temperament profiles might be influential in different areas of child behavior, influence scores in each of five areas measured (academics, sports, cultural trends, games, and inappropriate classroom behavior) were analyzed separately. In the area of academics, a significant effect for temperament was obtained (*F* = 9.04; *df* = 6; *p* < 0.001; *R*^2^ = 0.089) with Cluster 3 children (happy, social, active, and well self-regulated) having the highest influence score, and Cluster 6 (withdrawn, fearful, and low activity level) children having the least. There was a significant effect for influence in sports (*F* = 10.33; *df* = 6; *p* < 0.001; *R*^2^ = 0.067). Children in Cluster 5 (active, distractible, and negative) had the highest influence, and again children in Cluster 6 had the lowest. Temperament had a significant effect on influence regarding cultural trends (hairstyle, music, etc.; *F* = 11.76; *df* = 6; *p* < 0.001; *R*^2^ = 0.076) with children in Clusters 5, 2 (average with low negative emotionality), and 7 (withdrawn, low activity level, and low positive emotionality with low self-regulation) having the most and children in Cluster 6 having the least. Regarding make-believe games, there was a significant effect (*F* = 6.16; *df* = 6*; p* < 0.001; *R*^2^ = 0.038), but the effect was small. Only Cluster 5 children were distinct from the remaining clusters, and they had the most influence. By far the strongest effect of temperament on influence was on inappropriate classroom behavior (e.g., fooling around when the teacher was out of the classroom, talking back to the teacher; *F* = 30.41; *df* = 6; *p* < 0.001; *R*^2^ = 0.184). For these types of behaviors, children in Clusters 5 active, distractible, negative had the highest scores, and children in Clusters 6, 2, and 1 had the lowest. In summary, children who exhibited a high activity level, distractibility, and high negative emotionality (Cluster 5) were clearly the most influential children in these schools, while children who were socially withdrawn exhibited low levels of positive emotionality, and had a low activity level had the least influence on their peers.

**Table 4:** Cluster effects on peer nomination for total influence on peers.

Cluster	*n*	Homogeneous subgroup		
		1	2	3
6	48	−0.70		
2	146		−0.19	
1	322		−0.06	
7	37		−0.06	
3	99		0.11	
4	78		0.22	
5	58			0.97

Gabriel *post-hoc* test isolated statistically homogeneous subgroups of clusters.

### Association of temperament profiles with social status measures

One purpose of this research was to determine if dominance and/or prestige-related behaviors were characteristic of children who had different temperament profiles. Seven different measures of student status-related characteristics as perceived by peers were examined. A series of ANOVAs were calculated in which scores on each peer nominated status-related variable served as the dependent variable and cluster by grade level, cluster by gender, and cluster by minority/majority were entered separately as the independent variables. These results indicated the variance explained by cluster in all analyses explained three to four times the amount of variance explained by grade, gender, or minority/majority status. A small number of analyses resulted in significant main effects for the three demographic variables, and an even smaller number resulted in an interaction. Because the effects other than temperament explained less than 3.0% of the variance, these effects are not reported. Children in the three most influential cluster (5, 2, and Cluster 3) had a different blend of status-related attributes as viewed by their peers (see Table 5). Children in Clusters 2 and 3 are likely influential because they have skills (e.g., good at sports), valued attributes (e.g., tries hard at school), and interpersonal skills (e.g., sympathetic to peers) that contribute to being likeable and popular. Cluster 5 children who are the most influential are likely influential due to dominant, coercive behaviors (e.g., aggression) as well as being good at sports. Children in Cluster 6 were perceived as having the lowest social status of all six clusters and were the least influential.

**Table 5:** Mean social status descriptors by profile.

Profile	*n*	SPre^1^	Pop	Sym	Cool	Agg	Ath	Tries
1	313	0.07	−0.03	−0.21	0.00	0.00	−0.08	−0.28
2	144	**0.29** ^2^	−0.07	**0.31**	−0.22	−0.24	−0.08	**0.39**
3	99	**0.53**	**0.58**	**0.49**	0.11	−0.07	**0.48**	**0.84**
4	76	−0.09	**0.05**	**0.09**	0.19	0.00	**0.20**	0.01
5	54	−0.34	**0.20**	−0.23	**0.86**	**0.73**	**0.25**	−0.26
6	34	−0.47	−0.87	**0.01**	−0.58	−0.53	−0.56	−0.17
7	26	−0.80	−0.71	−0.53	−0.22	**0.65**	−0.32	−0.43
	Anova (eta)	0.087^3^	0.092	0.086	0.110	0.113	0.076	0.149

1 SPre, social preference; Pop, perceived popularity; Sym, shows sympathy; Cool, is cool, well know; Agg, is aggressive; Ath, is a good athlete; Tries, tries hard at school.

2 Means in bold are significantly different from means that are underlined. Means not in bold and underlined means are not significantly (*p* < 0.05) different from one another.

3 All cluster effects had a probability < 0.001.

### Marco-environmental effect on the association of peer influence and temperament profiles

Children who attended schools in two different kinds of social environments were examined in this research. While the environmental contexts were similar for the two groups of schools in many ways (e.g., lived in rural areas and had median family income significantly less than the state and national average), the educational attainment of the adult male population was different (i.e., persons 25 years and older). This was the result of the differences in educational attainment among minority populations. The ethnic/racial composition of the county in which Group B schools were located was predominantly White, while counties in which Group A schools were located had a much higher percentage of minority adults (about one-third of the population). The percentage of minority children in the public schools was even larger in Group A schools, constituting about 50% of the public-school population. The children in Group B schools who live in an environment comprised a more educated adult population might value different behavior characteristics than those in the Group A schools, where educational attainment among the adult population is more limited. This social environmental difference might create difference in the types of children who are viewed as most influential by their peers. To investigate this notion, the total influence scores of children were submitted to a multifactor ANOVA in which temperament cluster and school group were conceptualized as independent variables and the total influence score as the dependent variable. The results are reported in Table 6. This analysis resulted in a significant main effect for cluster (*F* = 9.67; *p* < 0.001), no main effect for School Group (*F* = 0.57; *p* = 0.45), but there was a significant Interaction (*F =* 3.53; *p* = 0.002). To determine if there were significant differences within profiles, a one-way ANOVA across school groups for each profile was calculated and summarized in Table 6. This resulted in a significant effect for Clusters 2 and 3, with children exhibiting this temperamental profile in school group B (i.e., more educated adult environment) having more influence on the peers than children exhibiting this profile in school group A (i.e., less educated adult environment). These children exhibited a status profile in which trying hard in school was an important factor along being sympathetic toward others and being likeable (having a high social preference score). Children in Cluster 5 (Active, distractible, and negatively emotional) did not have a significantly different influence score in the two school settings, although their total influence score was more than twice as high in the School A group (lower levels of adult education) than in School B (more educated adult population). Thus, it appears that the macro-environment in which the two groups of schools were situated had an effect on whether dominance had the greatest effect on peer influence (school group A) or prestige-related methods had the greatest effect on influence (school group B). To further analyze the differences between the two school groups, a similar analysis was conducted on each of the five areas of influence. This was done separately for Cluster 3 children who had the most status in School Group B, and Cluster 5 children that had high status in both school groups. As summarized in Table 7, Cluster 3 children had significantly more influence on academic issues (getting good grades, doing the homework) in school group B. But they also had more influence on youth culture in school group B than in school group A. Cluster 5 children had more influence on academics in school group A (less educated adult population), and also on the imaginary games children play.

**Table 6:** Effects of profile and school group on total influence on peers.

Profile	School group A			School group B			*p*	etasq
	*M*	SD	*N*	*M*	SD	*N*		
1	−0.01	(0.90)	177	−0.15	(0.86)	145	ns	0.006
2	−0.35	(0.75)	84	−0.02	(0.93)	62	0.03	0.032
3	−0.04	(0.89)	72	0.53	(1.11)	27	0.01	0.066
4	0.15	(0.88)	38	0.28	(0.92)	40	ns	0.006
5	1.25	(1.53)	34	0.56	(1.45)	22	ns	0.051
6	−0.74	(0.53)	19	−0.67	(0.53)	29	ns	0.005
7	−0.17	(0.75)	17	0.03	(1.16)	20	ns	0.011
Total			441			345		

**Table 7:** Comparison of influence of group 3 and group 5 on specific types of influence.

Influence type	Cluster	School group A			School group B			*p*	etasq
		*M*	SD	*N*	*M*	SD	*N*		
Good grade and homework									
	3	0.28	(1.17)	72	1.03	(1.44)	27	0.009	0.069
	5	0.56	(1.14)	34	−0.07	(0.90)	22	0.032	0.082
Sport									
	3	0.11	(1.04)	72	0.57	(1.26)	27	ns	0.034
	5	0.83	(1.45)	34	0.34	(1.12)	22	ns	0.032
Cultural Trends (hair style; clothes; music)									
	3	−0.12	(0.85)	72	0.35	(1.09)	27	0.026	0.050
	5	1.10	(1.54)	34	0.49	(1.50)	22	ns	0.038
Inappropriate behavior in the classroom									
	3	−0.30	(0.71)	72	−0.28	(0.43)	27	ns	0.000
	5	1.34	(0.34)	34	1.30	(2.05)	22	ns	0.000
Games									
	3	−0.01	(1.10)	72	0.13	(0.95)	27	ns	0.003
	5	0.95	(1.37)	34	0.27	(1.15)	22	0.050	0.066

## Discussion

Temperamental traits are important to psychological theory and in the practice of helping parents, teachers, and children because these traits can be observed very early in life and have been shown to relate to important outcomes for children throughout their developing years and even throughout the live span. These traits also have been shown to relate to various levels of the biology of the child (genes, the biochemistry of the nervous system, etc.). In the early stages of development of temperament theory, the biological underpinnings, particularly genetic influences, were viewed as one of the most important defining aspects of temperamental traits. As genetic research and its relationship to behavior and personality has progressed, it has become clear that almost all personality traits and behavioral responses have a genetic foundation (Shiner and Caspi, 2012; Plomin, 2018). Research on these traits would not have continued to grow as it has if the various traits typically thought of as being temperamental had not been demonstrated to be relatively stable (stability increasing with maturity; see Martin et al., 2020 for a review) and had they not been found to relate to behavior problems in childhood, diagnosed psychopathology, academic achievement, educational attainment in adulthood, and other important outcomes. But the focus on these guidepost outcomes in human life has not elucidated many of the social processes occurring in the life of the child that led to these outcomes. This is nowhere clearer than in the application of temperamental differences to children in schools, where the majority of research is on achievement and behavior problems. The research reported in this paper was designed to begin to fill one gap in our understanding of schooling; specifically, the influence students have on one another. Parents and teachers are aware that children who attend the same school have an influence on one another. The multi-billion-dollar industry of private schooling is to some extent built on this awareness. The awareness that children influence one another does not tell us which children are particularly influential, it does not tell us what areas of schooling are most impacted by peer influence (e.g., peer status, academic achievement, and inappropriate behavior in the classroom), and it does not address what individual differences of children lead to being influential. The research reported here is based on the hypothesis that individual differences in six temperamental traits has a substantial impact on influence processes in the classroom. This research is also based on the assumption that it is the configuration of these six traits considered together, rather than individual traits that will best illuminate how temperamental traits are related to peer influence in school. This assumption is based on research indicating that temperamental traits are correlated in complex and interactive ways. Research has demonstrated that temperamental traits are not highly stable. Correlations across 2-year periods, for example, typically vary from 0.40 to 0.70, but decline somewhat when longer retest intervals are used. Further, the impact of temperament in different social environments may be different. Thus, in the current context, it is important to determine what environmental factors affect change in how temperamental profiles are related to peer influence.

In this study of approximately 800 rural public-school children in 4th and 5th grades, it was determined that one group of children (Cluster 5) was perceived by peers as having the most influence. Of the seven clusters of children defined empirically by their temperament profiles (assessed by teachers), a relatively small group of children (7.3%) was found to have the most influence on their peers. Children in this cluster were viewed by their teachers as highly active, with above-average ratings on sociability, but exhibited high levels of negative emotionality and low levels of positive emotionality. They were also above average in distractibility. This group can be conceptualized as having low levels of self-regulation of emotion and attention. Notably, this cluster was also rated as being among the least inhibited and fearful of all temperament clusters. We investigated what areas of peer interaction this temperament group (Cluster 5) had most influence. They were among the most influential of all profiles in peer cultural trends (hair style, music preference, and peer language), and in what games were played with peers. They also had particularly strong influence on inappropriate behavior in the classroom (e.g., fooling around when the teacher left the room, talking back to the teacher). Their high activity level and distractibility, as well as their low level of fearfulness probably played an important role in their inappropriate behavior in the classroom. In addition to investigating which group of children was most influential, one aspect of this research investigated how temperamental profiles and influence was related to indicators of social status as assessed by peers. Peers perceived the children in Cluster 5 to be ‘cool’ more often than any other clusters and they had high scores on aggression. They were mildly above average in popularity and athletic skill. The influence of this group seemed to be based in part on their athleticism, on being socially aggressive, but also on their lack of inhibition and fearfulness. Perhaps most of all, they seem to be viewed as charismatic as indicated by being nominated frequently as ‘cool’. Thus, they can be thought of as using both domination and prestige forms of influence. Children who were perceived by peers as least influential across all five areas of school life were those belonging to Cluster 6 (6% of the sample). Their temperament profile was characterized by low activity level, low sociability, low levels of negative emotionality, and high inhibition/fearfulness. They had below-average scores on peer perceptions of likeability, popularity, trying hard at school, having sympathy for others, being cool, acting aggressively, and having athletic skill. Their lack of influence on others seemed to be a function of their withdrawal from social activities and being perceived as less skillful in sports. The two largest clusters (Clusters 1, 41.2%, and 2, 18.4%) were average on all temperamental characteristics, with Cluster 2 being viewed as of more negative mood Cluster 1. They also had near average scores on all types of influence based on peer nominations. Further, peer nominations of status-related characteristics were all in the average range as well, with Cluster 2 children being perceived as having moderately higher status than Cluster 1. One of the most important findings from this research was that the social milieu of the school had a significant effect on the influence exercised by the most influential groups of children. The aspect of the broader social environment that we focused on was the educational attainment of the adult population of the counties in which the children resided. Children in temperament Cluster 3 (happy, social, active, and, well self-regulated) were viewed as the second most influential group. When they lived in a county with higher adult educational attainment (particularly among adult males) they had more influence on academic behaviors (e.g., trying hard in school) than Cluster 3 who lived in rural counties with lower educational attainment. The reverse was true of children in Clusters 5. For these children, they had more influence when they lived in the counties with lower educational attainment.

### Theoretical implications

Temperament researchers, spurred on by findings of significant stability of temperament traits, as well as long-term significant prediction of adult behavior from measures obtained in early childhood, have made major strides in the understanding of child behavior. However, they have not paid much attention to environmental factors that may alter the expression of temperamental traits. The major exception to the rule is the role of parenting on temperamental characteristics (Bridgett et al., 2015; Bornstein et al., 2018). All temperament theorists and researchers posit that temperament is not static. While most behavioral characteristics understood as being rooted in temperament have been shown to have moderate stability, all data available indicate some children are very stable, most children exhibit some change, and a few children exhibit major changes in their trait level scores. What is less clear are the mechanisms and social forces that influence these changes. There is another type of change that is even less well understood; that is, how do children with the same temperamental profile alter their social behavior to meet changing environmental demands. The research reported here did not study change over time, but it does open the door to thinking about this question. We found that children with the same temperamental profile who live in different social environments engage that environment in different ways. Stated another way, those children who are influential in one environment are less influential in another. These findings remind temperament researchers that human beings are social animals and that temperamental characteristics may have a different impact in different social circumstances.

### Strengths, limitations, and future research

The research reported here utilizes measures of individual differences that have been shown to appear in the first few years of life (i.e., temperamental differences) to explore questions about which children have the most influence in the peer group in late elementary school. Temperamental individual differences were measured as individual traits based on teacher’s perceptions of their students. One of the strengths of this study is that temperament profiles have rarely been empirically developed based on teacher perception. These profiles were then used to investigate influence patterns that occur among peers in schools. A second strength of this research is that this is one of the first attempts to relate empirically derived temperament profiles to peer influence in a school setting. Further, the status characteristics of children as viewed by other children were studied in the context of temperament profiles, revealing that children with different temperament profiles manifest their influence through different sets of status-related characteristics (e.g., popularity) and behavior (e.g., showing sympathy). These associations will help researchers in the area social processes understand some links between individual differences, status, and influence. Finally, the research demonstrated that the broader social context in which children live is related to how and by whom peer influence is exhibited. These findings were strengthened by having independent measures of behavior from teachers and students. A strong point of the research is that each type of measurement (teacher rated temperament, student perceptions of influence, and student perceptions of social status-related behavior) were all measured in detail as well as globally. That is, six dimensions of temperament were assessed, influence was assessed in five areas of school life, and status was measured through global indices (e.g., perceived popularity) as well as specific behaviors related to related to likeability and social stature. The availability of these more specific aspects of influence and status allowed for the determination of what type of status and influence was most affected by temperamental differences. Finally, the sample size was large enough to allow for an application of a modeling technique that requires relatively large samples (latent profile analysis) and to allow for a model of seven different profile types (*n* = 797). Having a sample of this size in conjunction with a detailed assessment of student social lives from two perspectives (teacher and student) is very rare in the temperament literature. Despite these strengths, the research had several limitations. The data analyzed in the current study were obtained from teachers and children during one development period, and on one occasion. Thus, temperament, the timing of effects of temperament on both social status and temperament remain unclear. Further, temperament was assessed from the point of view of a one teacher in each classroom. The research would have been strengthened if more than one teacher assessed the temperament of each student. Parental assessment would have also enhanced the temperament assessment. In addition, there was a confound between the interpretation of the social environment, described at the county level, and the minority status of the participants and their families. This occurred because ethnicity/race and school group were entangled to some extent. The Group B schools were less diverse than the Group A schools. The findings would have been stronger if the diversity of the two school systems were similar. This type of design would have clarified the effects of educational attainment independent of other cultural factors that are associated with rural southern culture. A further weakness of this study was the reliance on county-level educational attainment data. The results would have been much clearer if the educational attainment of each individual family had been assessed. The findings reported here clearly indicate the need for a longitudinal approach in which temperamental traits are measured at several time periods in different environments to determine effects of the environment (a) on the measurements of traits over time, (b) on the association of temperament with social status phenomena, and (c) the effects of environments and developmental level on social influence patterns. To enhance the understand of temperamental effects on peer influence in different environment, special care to measure the environments as precisely as possible is critical.

## Data availability statement

The data analyzed in this study is subject to the following licenses/restrictions: The dataset was collected by the co-author and is still being analyzed for various publication. It is not available to the public. Requests to access these datasets should be directed to mlease@uga.edu.

## Ethics statement

The studies involving human participants were reviewed and approved by University of Georgia Ethics Review Board. Written informed consent to participate in this study was provided by the participants' legal guardian/next of kin.

## Author contributions

All authors listed have made a substantial, direct, and intellectual contribution to the work and approved it for publication.

## Conflict of interest

The authors declare that the research was conducted in the absence of any commercial or financial relationships that could be construed as a potential conflict of interest.

## Publisher’s note

All claims expressed in this article are solely those of the authors and do not necessarily represent those of their affiliated organizations, or those of the publisher, the editors and the reviewers. Any product that may be evaluated in this article, or claim that may be made by its manufacturer, is not guaranteed or endorsed by the publisher.
